# African swine fever virus remodels cellular host metabolism and promotes lipid droplet formation in macrophages and cell lines

**DOI:** 10.3389/fcimb.2026.1913777

**Published:** 2026-09-08

**Authors:** Inmaculada Galindo, Lucía Barrado-Gil, Miguel Ángel Cuesta-Geijo, Stuart D. Armstrong, Isabel García-Dorival, Ana Del Puerto, Jesús Urquiza, Paula Gil-Cortés, Covadonga Alonso

**Affiliations:** 1 Department of Biotechnology, Spanish National Research Council (CSIC), Instituto Nacional de Investigación y Tecnología Agraria y Alimentaria, Madrid, Spain; 2 Department of Infection Biology and Microbiomes, University of Liverpool, Liverpool, United Kingdom

**Keywords:** African swine fever virus, antivirals, enzyme inhibitors, lipid droplets, proteomics, transcriptome

## Abstract

**Background:**

African swine fever virus (ASFV) is a large cytoplasmic DNA virus that causes a highly lethal disease in pigs and poses a major threat to global animal health and food security. ASFV infection is characterized by extensive reprogramming of host cellular pathways to support viral replication, including immune regulation, stress responses, and metabolic processes. Increasing evidence indicates that lipid metabolism is actively modulated during infection; however, the functional contribution of specific lipid-related organelles to the ASFV replicative cycle remains to be fully defined.

**Methods:**

We characterized the host response to ASFV infection in primary porcine alveolar macrophages using Illumina-based RNA sequencing at two representative post-infection time points. Transcriptomic analyses were complemented by functional assays in Vero cells, employing pharmacological inhibitors of lipid droplet (LD) biogenesis, quantitative measurements of viral replication, infectivity, and gene expression, as well as proteomic profiling of purified LDs from infected cells.

**Results:**

ASFV infection induced profound changes in host gene expression, with significant enrichment of pathways involved in immune modulation, apoptosis, autophagy, and cellular metabolism. A remarkable finding was the marked upregulation of enzymes driving LD biogenesis. Functional studies showed that LDs were dynamically remodeled during infection, increasing in abundance and redistributing toward viral replication factories. Proteomic analysis of LDs from infected macrophages identified both structural and non-structural ASFV proteins, including subunits of the viral transcription machinery. Accordingly, pharmacological disruption of LD formation pathway prominently reduced viral genome replication, viral protein synthesis, infectivity, and virus production, exceeding 95% in some conditions.

**Conclusions:**

These findings establish lipid droplets as central host organelles that support efficient ASFV replication and reveal a tight physical and functional coupling between viral replication and host lipid metabolism. Targeting LD biogenesis represents a promising host-directed antiviral strategy against ASFV.

## 1 Introduction

Viruses rely on host cellular machinery to support their replication and propagation. As part of their strategy to evade host defenses and prioritize viral protein production, many viruses manipulate host protein synthesis, thereby suppressing antiviral responses and establishing an environment conductive to replication while limiting immune detection.

African swine fever (ASF) is a highly contagious and often fatal hemorrhagic disease affecting domestic and wild swine, for which no effective vaccine or antiviral therapy is currently available. Originally endemic in Africa, ASF has spread over the past decade throughout Asia and Europe, reaching a global distribution with major economic consequences. Several European countries, including Italy, are currently affected, and recent reports have confirmed its presence in wild boars in Catalonia, Spain (WOAH) (WOAH (World Organisation for Animal Health), 2025).

The causative agent, African swine fever virus (ASFV), belongs to the *Asfarviridae* family (Dixon et al., 2005). ASFV is a large double-stranded DNA virus with a complex structural organization and a cytoplasmic site of replication. Its primary targets are porcine macrophages and monocytes (Malmquist and Hay, 1960; Baek et al., 2022; Droesbeke et al., 2025; Chu et al., 2024).

ASFV gene expression follows a temporally regulated cascade comprising immediate-early, early, intermediate, and late genes, each controlled by distinct promoter elements and stage-specific transcription factors. Immediate-early and early genes are expressed prior to viral DNA replication, whereas intermediate and late genes are transcribed post-replication. Essential enzymes required for viral replication are rapidly synthesized upon infection by virion-packaged transcriptional components that mediate early mRNA synthesis (Salas et al., 1981; Almazan et al., 1992, 1993; Rodriguez et al., 1996; Dixon, 2025). This early expression program enables ASFV to modulate host pathogen responses from the initial stages following infection, however, the full spectrum of host cellular processes altered by ASFV remains poorly understood.

To investigate host transcriptional responses during ASFV infection, we performed RNA sequencing (RNA-seq) on porcine macrophages at different time points post-infection corresponding to the early infection, before replication and, the later phase of active viral replication and assembly. Transcriptomic profiling revealed modulation of immune-related pathways, including apoptosis and autophagy. Strikingly, we observed a marked upregulation of genes involved in LD biogenesis, prompting further investigation into the roles and subcellular dynamics of these organelles. ASFV is known to exploit the cholesterol (CHOL) pathway of host lipid metabolism and reorganizes membrane systems to build cytoplasmic viral replication factories (VFs). During infection, the virus hijacks endosomal membranes and redirects CHOL trafficking, increasing the cytoplasmic pool of free CHOL and targeting it to VFs. Accordingly, pharmacological inhibitors of CHOL trafficking, such as itraconazole and 25-hydroxycholesterol, disrupt VF formation and impair ASFV replication (Cuesta-Geijo et al., 2015, 2017; Galindo et al., 2019). In addition, many viruses reprogram host lipid metabolism by inducing LD biogenesis and remodeling LD dynamics, thereby providing energy sources, lipid precursors, and cellular platforms that support efficient viral replication and pathogenesis (Herrera-Moro Huitron et al., 2023).

LDs are dynamic cytoplasmic organelles composed of a hydrophobic core of neutral lipids, primarily triacylglycerols (TAGs) and CHOL esters (CEs), surrounded by a phospholipid monolayer embedded with proteins involved in lipid metabolism, membrane trafficking, and signaling (Zhang and Liu, 2019). LDs originate at specific sites within the endoplasmic reticulum (ER), where neutral lipids accumulate between membrane bilayers, generating nascent droplets that bud from the ER in a process regulated by seipin, which plays an essential role in this process. TAG synthesis proceeds via a four-step enzymatic cascade involving glycerol-3-phosphate O-acyltransferase (GPAT), 1-acylglycerol-3-phosphate O-acyltransferase (AGPAT), phosphatidic acid phosphatase (PAP/lipin), and diacylglycerol O-acyltransferases (DGATs). These reactions require activated fatty acids, which are esterified with coenzyme A (CoA) by acyl-CoA synthetases (ACS) in an ATP-dependent manner. CHOL esterification, another essential component of LD formation, depends on *de novo* CHOL biosynthesis, initiated by the generation of acetyl-CoA from acetate or citrate. CHOL is subsequently esterified by acyl-CoA:cholesterol O-acyltransferases (ACATs) to produce CEs, which are then incorporated into LDs (Farias et al., 2022). In addition, LD-associated proteins such as the interferon-inducible antiviral factor Viperin and enzymes involved in cholesterol biosynthesis, including HSD17B7, have been shown to localize to or functionally interact with these organelles, linking lipid droplet dynamics to host antiviral responses and sterol metabolism.

In this study, we investigated the molecular interplay between lipid metabolism and viral pathogenesis in the context of the extensive cellular and metabolic reprogramming induced by the virus and the functional role of LD biogenesis during ASFV infection. Understanding these host-virus interactions may reveal novel targets for antiviral intervention and provide broader insight into ASFV biology.

## 2 Material and methods

### 2.1 Cell culture and viruses

Swine alveolar macrophages were collected by alveolar lavage with phosphate-buffered saline (PBS) as previously described (Carrascosa et al., 1982) and cultured at 37°C and 5% CO_2_ in RPMI medium containing inactivated 10% swine serum, 2 mM l-glutamine, 50 μM 2-mercaptoethanol, 20 mM Hepes and 30 μg/ml gentamycin. Vero cells (CCL-81; renal fibroblasts) were obtained from the American Type Culture Collection (ATCC) and cultured at 37°C and 5% CO_2_ in Dulbecco’s modified Eagle’s medium (DMEM) supplemented with 100 IU/ml penicillin, 100 μg/ml streptomycin, 2 mM L-glutamine, and 5% heat-inactivated fetal bovine serum (FBS) which was reduced to 2% during viral infection.

For infections, all experiments were performed in triplicates using the cell-adapted ASFV isolate BA71V and its derived recombinants viruses. Virus stocks were titrated as previously described (Enjuanes et al., 1976). Purified ASFV was prepared by ultracentrifugation at 40,000 x *g* through a 40% (w/v) sucrose cushion in PBS for 50 min at 4°C. Vero cells were infected at a multiplicity of infection (MOI) of 1 PFU/cell.

Alveolar porcine macrophages were infected at a multiplicity of infection (MOI) of approximately 100 PFU/cell. In this case, virus adsorption was carried out by spinoculation as previously described (Carter et al., 2005).

For flow cytometry analyses, the recombinant viruses BPP30GFP and B54GFP were used. BPP30GFP expresses green fluorescent protein (GFP) under the control of the promoter of the early viral protein p30 (Barrado-Gil et al., 2017) whereas B54GFP expresses GFP as a fusion protein of the viral p54 protein. For confocal microscopy analyses, recombinant viruses B54GFP and B54mCherry were employed, which express GFP and mCherry, respectively, as fusion proteins of the viral p54 protein, an early/late protein that mainly accumulates at viral replication sites (Alonso et al., 2013; Hernaez et al., 2006).

### 2.2 Reagents and cell viability determination

Triacsin C (TC) was purchased from Enzo, FSG67 from MedChemExpress and Oleic acid (OA), A922500 and CI-976 and from Sigma Aldrich. Stock solutions were dissolved in DMSO and working solutions were determined by cytotoxicity assays. Vero cells were seeded in 96-well plates and incubated with DMEM supplemented with 2% FBS containing each compound at concentrations ranging from 0 to 100 μM. After 24 h, cell viability was measured by Cell Titer 96 AQueous Non-Radioactive Cell Proliferation Assay (Promega) following the manufacturer’s instructions. Cell viability was reported as the percentage of absorbance in treated cells relative to DMSO treated cells. The 50% cytotoxic concentration (CC50) was calculated and non-toxic working concentrations were chosen to test the effect of these compounds. The estimation of the half maximal inhibitory concentration (IC50) was performed in those reagents with at least 50% efficacy and a dose-dependent inhibition of the infection. The values of the IC50 were calculated by nonlinear regression analysis of dose–response curves using the GraphPad Prism software. All experiments using these compounds followed the same procedure, with cells pretreated for 1 h prior to infection and treatments maintained throughout the infection cycle at previously established non-toxic concentrations.

### 2.3 RNA extraction, library preparation and Illumina sequencing

Alveolar macrophages were infected with the BA71V strain at a MOI of approximately 100 PFU/cell. Viral adsorption was facilitated by spinoculation, after which the cells were incubated at 37 °C for either 6- or 16-hours post-infection. Total cellular RNA from three independent biological replicates was extracted using TRI Reagent (Sigma-Aldrich) following the manufacturer’s protocol and subsequently quantified with a NanoDrop ND-1000 spectrophotometer (Thermo Scientific). Libraries were prepared according to the instructions of “NEBNext Ultra Directional RNA Library Prep kit for Illumina” (New England Biolabs), following the protocol “Poly (A) mRNA Magnetic Isolation Module”. The input amount of total RNA to start the protocols was >300 ng as quantified by an Agilent 2100 Bioanalyzer using a RNA6000 nano LabChip kit. The fragmentation time used was 15 min. We performed the library amplification included in the cited protocol using 13 PCR cycles.

The obtained libraries were validated and quantified by an Agilent 2100 Bioanalyzer using a DNA7500 LabChip kit and an equimolecular pool of libraries titrated by qPCR using Kapa-SYBR FAST qPCR kit forLightCycler480 (Kapa BioSystems) and a reference standard. The pool of libraries was denatured prior to be seeded on a flowcell at 2.2 pM density, where clusters were formed and sequenced using a “NextSeq™ 500 HighOutput Kit”, in a 1x75 single read sequencing run on a NextSeq500 sequencer. Approximately 20–25 million pass-filter reads were produced for each sample, which were used for further bioinformatics analysis.

### 2.4 Bioinformatic analysis: preprocessing, mapping and differential expression analysis

The quality of the obtained fastq was assessed using FastQC tool version 0.11.5 (https://www.bioinformatics.babraham.ac.uk/projects/fastqc/). The fastq files were Prinseq version 0.18.2 (Schmieder and Edwards, 2011) used to pre-process removing low-quality sequences (phred < 28), reads shorter than 50bp, and Ns content (rejecting reads over 5% of Ns and removing terminal Ns) and artefacts. This preprocess was carried out in a pipeline integrated in the software package GPRO (Futami et al., 2011).

Mapping of RNAseq Reads were performed using TopHat v2.1.1 and differential expression analysis were performed using the Cufflinks package v2.2.1 (Trapnell et al., 2012). For mapping as reference the *Sus scrofa* genome version Sscrofa10.2 (ftp.ensembl.org/pub) and as annotation file Sus_scrofa.Sscrofa10.2.86.gtf were used. The obtained alignment files were processed and levels of expression with p-value < 0.05 were considered as significant differential expression, and a 2-fold variance was used to identify the genes differentially expressed between conditions. The analysis and visualization of the data was carried out using the R package cummeRbund (Goff et al., 2013).

Gene Ontology Enrichment Analyses was performed using GOseq package in R (Young et al., 2010) implemented in GPro Suite (Futami et al., 2011). GOseq was developed specifically to account for transcript length bias in GO analyses based on RNA-seq data. For each comparison, upregulated and downregulated gene sets (no fold change cutoff) were input separately into GOseq. A P value cutoff of 0.05 was used.

Finally, we performed a metabolic analysis of the highly relevant differentially expressed genes recovered by retrieving information from the different metabolic pathways by downloading KEGG maps (Kotera et al., 2012) using the enzymatic codes associated with GO functional categories.

For the analysis of the virus transcriptome Reads were mapped using the Bowtie2 v2.2.5 (Langmead et al., 2009), Samtools v.1.2 (Li et al., 2009) was used to produce the alignment in bam format. The step abundance estimation of the reads for each virial transcript was performed via Corset v1.06 (Davidson and Oshlack, 2014). Differential expression between infection times was determined using the numbers of reads mapped to the assayed genes as inputs for EdgeR (Robinson et al., 2010). Each transcript showing a significate fold change with a false discovery rate of 5% or lower was identified as differentially expressed.

Statistical analysis and graphical representations (Venn analysis and heatmaps) were performed using the statistical package of GPRO software (Futami et al., 2011).

### 2.5 RNA preparation and RT-qPCR analysis for mRNA expression

Total RNA from macrophages and Vero cultured cells was isolated with TRI-Reagent (Sigma-Aldrich), following the manufacturer’s recommendations. cDNA was synthesized using QuantiTect Reverse Trancription kit (Qiagen) following the manufacturer’s instructions. cDNA levels were quantified using NanoDrop spectrophotometer. Primer set used for 18S gene is available from Qiagen (QuantiTect Primer Assay) and gene-specific qPCR primers designed using primer3Plus (http://www.bioinformatics.nl/cgi-bin/primer3plus/primer3plus.cgi/). Amplification was performed using ABI 7500 Fast RT-PCR System (Applied Biosystems) using a QuantiTect SYBR Green PCR kit (Qiagen) and following manufacturer’s recommendations. Fold change was determined using the 2^−ΔΔC(t)^ method of relative quantification with GAPDH or 18S as the endogenous control for normalization (Livak and Schmittgen, 2001) and melting curves were used to verify the specificities of products. The primers used are detailed in the supplementary figure.

### 2.6 Fluorescence and confocal microscopy

Cells were grown on glass coverslips and fixed in 4% paraformaldehyde (PFA) in PBS for 12 min and permeabilized with PBS containing 0.1% Saponin and 0.5% BSA for 1h. Nuclei were stained with SYTOX Deep Red Nucleic Acid Stain (Invitrogen) before mounting onto glass slides using ProLong Gold (Invitrogen) and imaging with a TCS SPE confocal microscope.

Image analyses were performed using Leica Application Suite advanced fluorescence (LAS AF Lite) (Leica Microsystems) and ImageJ software, including the JACoP plugin for colocalization analysis.

### 2.7 Lipid droplets staining

Specific probes for LDs were HCS LipidTOX Red Neutral Lipid Stain (Fisher Scientific) and BODIPY 493/503 (Molecular Probes). Vero cells grown on coverslips were rinsed with PBS, fixed in 4% PFA, washed, and incubated with LipidTOX at a 1:200 dilution in PBS for 30 min. BODIPY 493/503 was added directly to the cells at a final concentration of 2 μM in PBS and incubated for 15 min at 37°C. Then, cells were fixed in 4% PFA and mounted on glass slides for confocal microscopy analysis or trypsinized to generate a cell suspension for flow cytometry studies. As positive control of BODIPY staining and LD detection, we treated Vero cells with Oleic acid (100 μM) during 16 hours. LDs were quantified from BODIPY-stained confocal images using ImageJ software. LDs were identified by threshold-based segmentation of BODIPY-positive structures. The number of LDs per cell and the mean LD area (µm²) were determined using the Analyze Particles function.

### 2.8 Flow cytometry analysis

For detection of ASFV, Vero cells were pretreated with inhibitors at the indicated concentrations in growth medium for 1 h at 37°C followed by infection with recombinant ASFV BPP30GFP or B54GFP at a MOI of 1 PFU/cell for 16 h. Cells were washed with PBS, harvested by trypsinization, washed and collected in flow cytometry buffer (PBS, 0.01% sodium azide, and 0.1% BSA). In order to determine the percentage of infected cells per condition, 10,000 cells/time point were scored using a FACS Canto II flow cytometer (BDSciences) and analyzed using the FlowJo software. Infected cell percentages obtained after compounds treatments were normalized to values found in control samples.

Quantification of LDs was also performed by flow cytometry as described previously (Qiu and Simon, 2016). Cells were incubated with 2 μM BODIPY 493/503 staining solution in PBS for 15 min at 37°C. Then, cells were pelleted at 250 x *g*, 5 min, 4 °C. Supernatant was aspirated and the cell pellet washed with PBS and resuspended in flow cytometry buffer. We acquired 10,000 events per condition analyzed by mean fluorescence.

### 2.9 Acyl-CoA synthetase activity measurement

Vero cells were seeded in six-well plates, infected with ASFV a MOI of 1 and harvested at the indicated times. Acyl-CoA Synthetase was quantified using the Acyl-CoA Synthetase Assay Fluorometric Kit (Abcam) following manufacturer guide. Fluorescence was measured in an EnSight Multimode Plate Reader (PerkinElmer) with excitation at 535 nm and emission at 587 nm. Data were normalized to the activity of the lysate from mock-infected cells.

### 2.10 Western blotting

Equivalent amounts of protein lysates were electrophoresed in sodium dodecyl sulfate polyacrylamide gels (SDS-PAGE) and transferred to a nitrocellulose membrane (Bio-Rad). Nonspecific antibody binding sites were blocked with 5% skimmed milk powder in PBS-0.05% Tween-20 for 1 h and then incubated with anti-p30 monoclonal antibody 1:1000 (kindly given by Dr. Escribano, INIA), anti-p72 monoclonal antibody, clone 1BC11 (Ingenasa) 1:2000, anti-calnexin (Abcam ab22595) 1:1000, polyclonal serum against the viral protein UBCv1 (Barrado-Gil et al., 2020) 1:2000, polyclonal serum against the viral protein 54 (obtained in the laboratory) 1:500, anti-Perilipin 3/TIP47 (Abcam) 1:1000 or anti-tubulin (Sigma) 1:2000. Finally, immunoblot was incubated with suitable horseradish peroxidase-conjugated secondary antibodies. Protein expression was analysed by chemiluminiscence using the molecular imager Chemidoc XRS plus Imaging System. Band densitometry was performed with Image Lab software (BioRad) and data normalized to control values.

### 2.11 Detection of viral DNA by quantitative PCR

DNA from cells treated with the indicated concentration of inhibitors and infected with ASFV at a MOI of 1 PFU/cell for 16 hpi in triplicates, was purified using “Dneasy blood and tissue kit” (Qiagen) following the manufacturer’s instructions. DNA concentration was measured using a Nanodrop spectrophotometer. Uninfected cells and cells infected in the absence of drug were used as controls. The PCR assay used fluorescent hybridization probes to amplify a region of the p72 viral gene, as described previously (King et al., 2003). DNA was extracted from the ASFV viral stock, and serial dilutions were prepared to generate a standard curve for the absolute quantification of viral DNA. Reactions were performed using the ABI 7500 Fast Real-Time PCR System (Applied Biosystems) with the following parameters: 94 °C for 10 min and 45 cycles of 94 °C for 10 s and 58 °C for 60 s.

### 2.12 Purification of lipid droplets

Vero cells were seeded in T150 culture flasks and infected with ASFV at a MOI of 1 PFU/cell for 16hpi. LDs were isolated by flotation using the Abcam lipid droplet isolation kit (ab242290) according to the manufacturer’s instructions. Six sequential 270µl fractions were collected from the top of the gradient. LD fraction purity was evaluated by Western blot using antibodies against markers of distinct subcellular compartments. Purified LDs from each replicate were subjected to liquid chromatography–mass spectrometry (LC–MS) to characterize the LD-associated proteome during ASFV infection.

### 2.13 Proteomics analysis

SDS-PAGE (10% polyacrylamide) was run until the whole samples penetrated in the resolving gel (ca. 1 cm of total migration). Gels were stained with Colloidal Blue Staining Kit (Invitrogen). Whole proteomes were excised and cut in small pieces prior to in-gel digestion with trypsin, and destained with 50 mM ammonium bicarbonate (ABC), (Sigma-Aldrich) and 50% acetonitrile (ACN), (Fisher Chemical). Samples were then reduced with 10 mM dithiothreitol (Bio-Rad) in 50 mM ABC, and alkylated with 55 mM iodoacetamide (GE Healthcare Life Sciences) in 50 mM ABC. Then, gel pieces were digested with porcine trypsin (Thermo Fisher Scientific), at a final concentration 12.5 ng/ml in 50 mM ABC, overnight at 37 °C. Peptides were extracted using 100% ACN and 0.5% trifluoroacetic acid, (Sigma-Aldrich), purified using a ZipTip (Millipore, Sigma-Aldrich), and dried. Finally, samples were reconstituted in 0.1% formic acid before analysis by nano system liquid chromatography-tandem mass spectrometry (nLC–MS/MS).

Peptide separations were carried out on an Easy-nLC 1000 nano system (Thermo Scientific). For the analysis, samples were loaded into a precolumn Acclaim PepMap 100 (Thermo Scientific) and eluted in a RSLC PepMap C18, 50 cm long, 75 µm inner diameter and 2 µm particle size (Thermo Scientific). The mobile phase flow rate was 300 nL/min using 0.1% formic acid in water (solvent A) and 0.1% formic acid and 100% acetonitrile (solvent B). The gradient profile was set as follows: 5-35% solvent B for 100 min, 35%-45% solvent B 20 min, 45%-100% solvent B 5 min, and 100% solvent B 15 min. Four microliters (about 1 µg) of each sample were injected. MS analysis was performed using a Q-Exactive mass spectrometer (Thermo Scientific). For ionization, 1900 V of liquid junction voltage and 300 °C capillary temperature was used. The full scan method employed a m/z 300–1800 mass selection, an Orbitrap resolution of 70,000 (at m/z 200), a target automatic gain control (AGC) value of 3e6, and maximum injection times of 100 ms. After the survey scan, the 15 most intense precursor ions were selected for MS/MS fragmentation. Fragmentation was performed with a normalized collision energy of 27 eV and MS/MS scans were acquired with a starting mass of m/z 200, AGC target was 2e5, resolution of 17500 (at m/z 200), intensity threshold of 8e4, isolation window of 2.0 m/z units and maximum IT was 100 ms. Charge state screening was enabled to reject unassigned, singly charged, and equal or more than seven protonated ions. A dynamic exclusion time of 30s was used to discriminate against previously selected ions.

Protein identification by nLC-MS/MS was performed at the Proteomics and Genomics Facility (CIB-CSIC) and by our collaborators at University of Liverpool (UoL). MS data were analysed with MaxQuant (version 2.4.7) with default Orbitrap workflows. Raw spectral files were searched against UniProtKB proteomes UP000029965 (Chlorocebus sabaeus; 19–143 sequences) and UP000000624 (African swine fever virus; 159 sequences). The false discovery rate (FDR) was set to 0.01 for both peptide and protein identifications.

### 2.14 Statistical analysis

Statistical analysis was performed using GraphPad Prism 6. One-way ANOVA was used, followed by Bonferroni’s *post hoc* test for multiple comparisons. Data are presented as mean +- SD from at least three independent experiments. A p-value < 0.05 was considered statistically significant.

Data from LC-MS studies were analyzed by using Perseus v1.6.15.0 software (Max Planck Institute, Munich, Germany). The selected proteins identified were visualized using Metascape software (v3.5.20240101; http://metascape.org). To elucidate the functional roles of the identified proteins, several analyses were performed, including pathway and process enrichment analysis. All experiments were performed using three independent biological replicates.

## 3 Results

### 3.1 RNAseq study based on the transcriptome analysis

Transcriptomic analysis of porcine macrophages was conducted using next-generation sequencing to compare transcripts from uninfected control macrophages with those infected with ASFV at 6- and 16-hours post-infection (hpi). These time points corresponded to the early phase of infection and to the period of active viral replication and assembly, respectively. Raw sequencing reads were used to generate a transcriptome assembly, enabling the identification of differentially expressed and regulated genes and transcripts. Sample quality control was performed by assessing RNA concentration, fragment size distribution, and integrity using on-chip electrophoresis.

Differential expression analyses were carried out for the following comparisons in triplicates: (a) control vs. infected macrophages at 6 hpi, (b) control vs. infected macrophages at 16 hpi, and (c) macrophages infected at 6 hpi vs. 16 hpi. The sequencing process generated approximately 200 × 10^6^ short reads, with an average of 20 × 10^6^ reads per sample. From these, 85% of reads from control samples aligned to Sus scrofa genes. The proportion of cellular mRNAs obtained ranged from 39% to 58% of total reads at 6 and 16 hpi, respectively.

**Figure 1:**
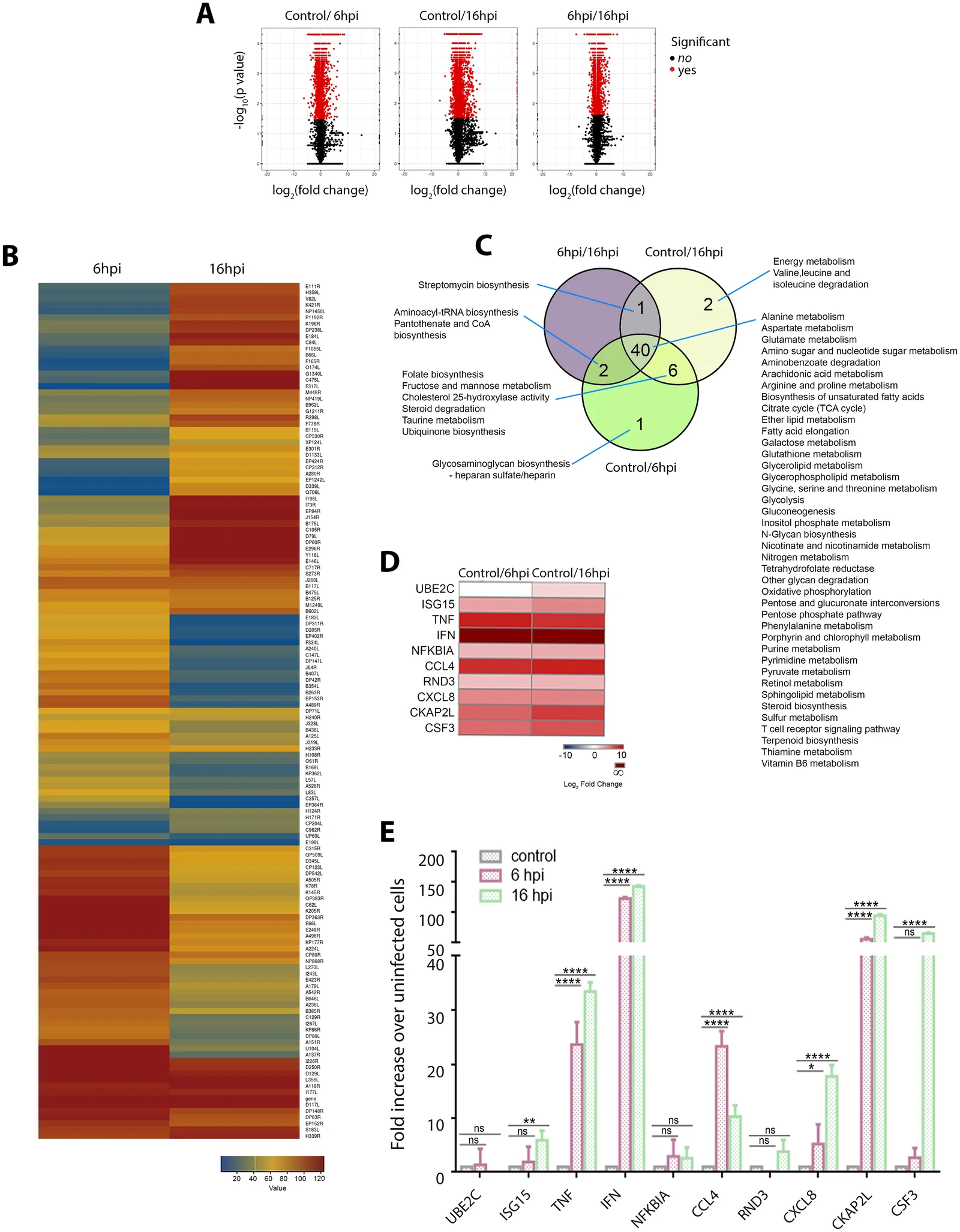
Transcriptomic analysis of infected macrophages at 6 and 16 hours post-infection (hpi). **(A)** Volcano plots showing gene expression comparisons based on log_10_ *p*-value versus log_2_ fold change of differentially expressed genes; statistically significant genes are highlighted in red. **(B)** Heat map of viral transcripts that were significantly differentially expressed at 6 and 16 hpi. The color key indicates relative expression levels, ranging from red (high abundance) to blue (low abundance). **(C)** Venn diagram illustrating the relationships among the three transcriptomes. **(D)** Heat map of 10 representative genes selected from significantly differentially expressed cellular transcripts. **(E)** Expression levels of selected genes were validated by RT-qPCR. The GAPDH gene was used as a reference for data normalization. Expression values are shown as fold change relative to uninfected macrophages. Asterisks denote statistically significant differences (*p < 0.05; **p < 0.01; ****p < 0.0001). Colors in the heatmaps represent the mean log_2_ fold change values calculated from three biological replicates per sample.

After normalization and differential expression analysis, a total of 18,549 significantly differentially expressed genes were identified in macrophages (red dots in volcano plots; **Figure 1A**). At 6 hpi, 7,127 genes were differentially expressed (2,745 O and 4,382 D). The comparison between 6 and 16 hpi revealed 4,509 differentially expressed genes (2,516 O and 1,993 D). At 16 hpi, 6,913 genes were differentially expressed, including 3,239 overexpressed (O) and 3,674 downregulated (D) transcripts. The proportion of sequencing reads mapping to the viral transcriptome was higher at 6 hpi, likely as a result of increased RNA degradation observed at later stages of infection (16 hpi), as indicated by electrophoretic profiles (data not shown).

Normalized expression data were visualized in heat maps, which displayed ASFV genes expressed in their characteristic sequential manner (**Figure 1B**). Relationships among the three transcriptomes, as well as significant alterations in metabolic pathways, were summarized in a Venn diagram. Relevant pathways included multiple branches of lipid metabolism, steroid degradation, cholesterol 25-hydroxylase activity, inositol phosphate metabolism, and the T-cell receptor signaling pathway (**Figure 1C**).

### 3.2 Validation of transcriptome analysis

Changes in the transcription of 10 selected cellular genes identified by the NGS analysis (**Figure 1D**) were validated by quantitative real-time reverse-transcription PCR in ASFV-infected macrophages (**Figure 1E**). Real-time PCR (RT-PCR) corroborated the upregulation observed in the sequencing data, confirming a strong overexpression of transcripts such as IFN (interferon alpha-omega) and TNF (tumor necrosis factor), as well as other genes exhibiting significant but moderate fold changes including CXCL8 (C-X-C motif chemokine ligand 8; IL-8), CCL4 (C-C motif chemokine ligand 4), CKAP2L (cytoskeleton-associated protein 2-like), CSF3 (granulocyte colony-stimulating factor), and ISG15 (ubiquitin-like interferon-stimulated gene 15). These transcriptional changes are consistent with the pathogenesis of ASFV infection and correlate well with the cytokine secretion, inflammatory responses, and febrile clinical signs of the disease. Some transcripts were found to be overexpressed only at specific time points, but not at later stages of infection.

### 3.3 Analysis of transcripts related to apoptotic process, autophagy and lipid droplets

**Figure 2:**
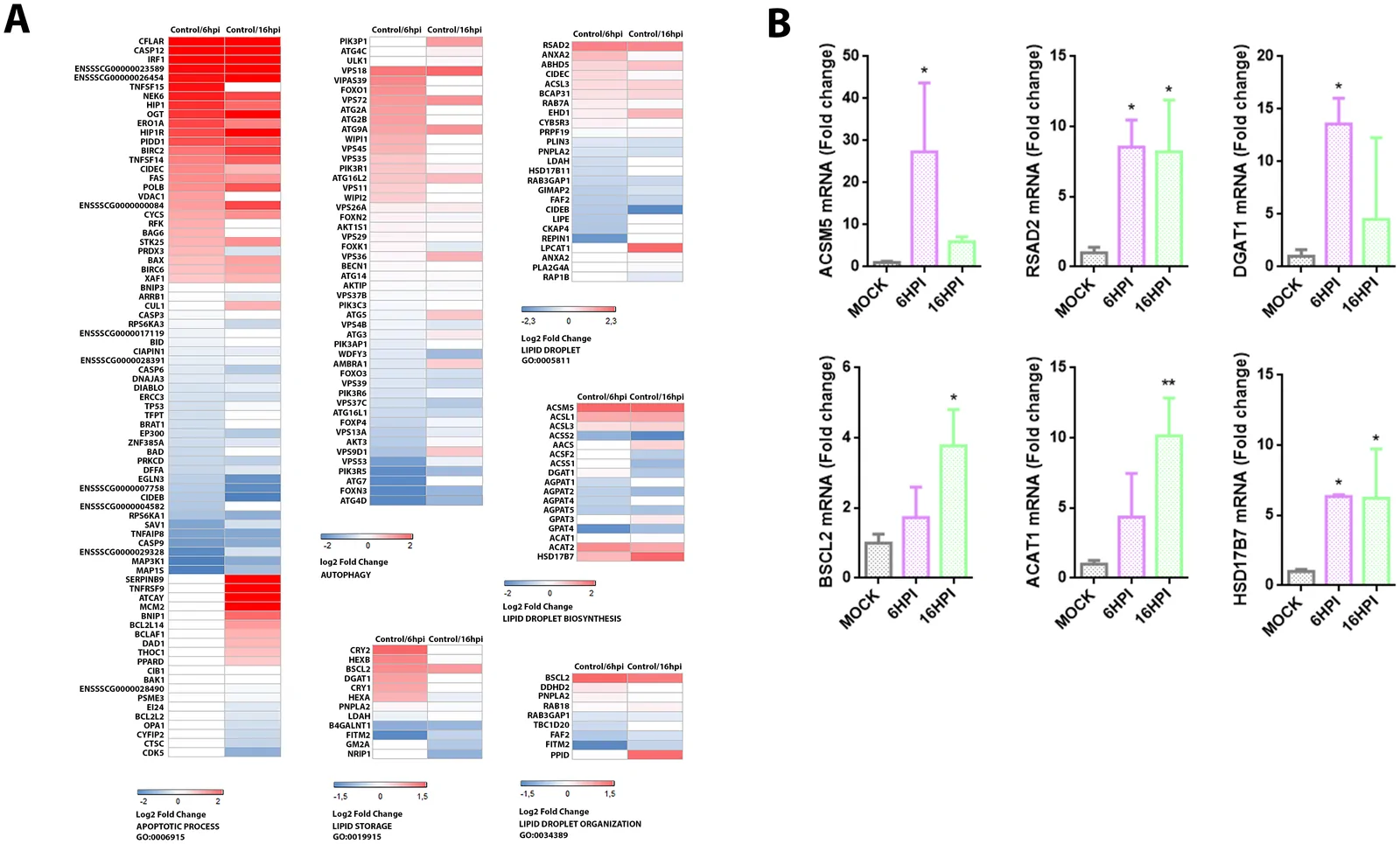
**(A)** Heat map of transcripts that were significantly differentially expressed in infected macrophages at 6 and 16 hpi. Colors in the heatmaps represent the mean log_2_ fold change values calculated from three biological replicates per sample. The color key below the heat map indicates the relationship between color and expression level, ranging from red (high abundance) to blue (low abundance). **(B)** Expression levels of the indicated genes involved in the LD biosynthesis pathway were assessed by RT-qPCR in infected Vero cells at 6 and 16 hpi. Quantified mRNA levels were normalized to 18S ribosomal RNA and to mock-infected control values. Data are presented as mean ± SD from three independent experiments. Asterisks indicate statistically significant differences (*p < 0.05; **p < 0.01).

Transcriptomic analysis revealed the involvement of apoptosis, autophagy, and notably several lipid-related pathways in ASFV-infected macrophages (**Figure 2A**). Among these pathways, the analysis highlighted a previously underappreciated category: LD biogenesis. Therefore, transcripts associated to this pathway were selected for further examination. The differentially expressed transcripts included ACAT1 (cholesterol acetyltransferase 1), DGAT1 (diacylglycerol O-acyltransferase 1), ACSM5 (acyl-CoA synthetase medium-chain family member 5), and BSCL2 (seipin), a key factor associated with LD formation. Overexpression of RSAD2 was also detected; this gene encodes viperin, an interferon-inducible antiviral protein localized to the endoplasmic reticulum and LDs. Additionally, increased HSD17B7 (hydroxysteroid 17-beta dehydrogenase 7), a CHOL biosynthetic enzyme, was observed upon infection.

RT-qPCR was used to validate transcript abundance in ASFV-infected Vero cell line at 6 and 16 hpi, using 18S rRNA as the internal control (**Figure 2B**). Primer sequences are provided in **Supplementary Figure 1**. RT-qPCR confirmed the marked upregulation of key lipid-metabolic transcripts identified by transcriptomics, including ACSM5, RSAD2, DGAT1, BSCL2, ACAT1, and HSD17B7. A pronounced early increase (6 hpi) was detected for ACSM5 (which catalyzes the activation of fatty acids by CoA to form acyl-CoA, an initial step in fatty acid metabolism) and for DGAT1, which catalyzes the conversion of diacylglycerol and fatty acyl-CoA into triacylglycerol, essential for lipid droplet formation. In contrast, upregulation of ACAT1, the enzyme responsible for intracellular CHOL esterification, was detected at later time points (16 hpi). A similar late phase overexpression pattern was observed for BSCL2, which determines the sites of LD formation at the endoplasmic reticulum (Chung et al., 2019). RSAD2 (viperin) and HSD17B7 were upregulated at both early and late stages of infection. A subset of hydrosteroid dehydrogenases (HSD) have been found to be LD-associated proteins (Liang et al., 2025).

Taken together, these findings indicate that the LD biogenesis pathway is strongly regulated at the transcriptional level during ASFV infection, showing comparable behavior in both primary macrophages and Vero cells, thereby supporting the use of Vero cells for subsequent experiments.

### 3.4 Lipid droplets increased upon ASFV infection

**Figure 3:**
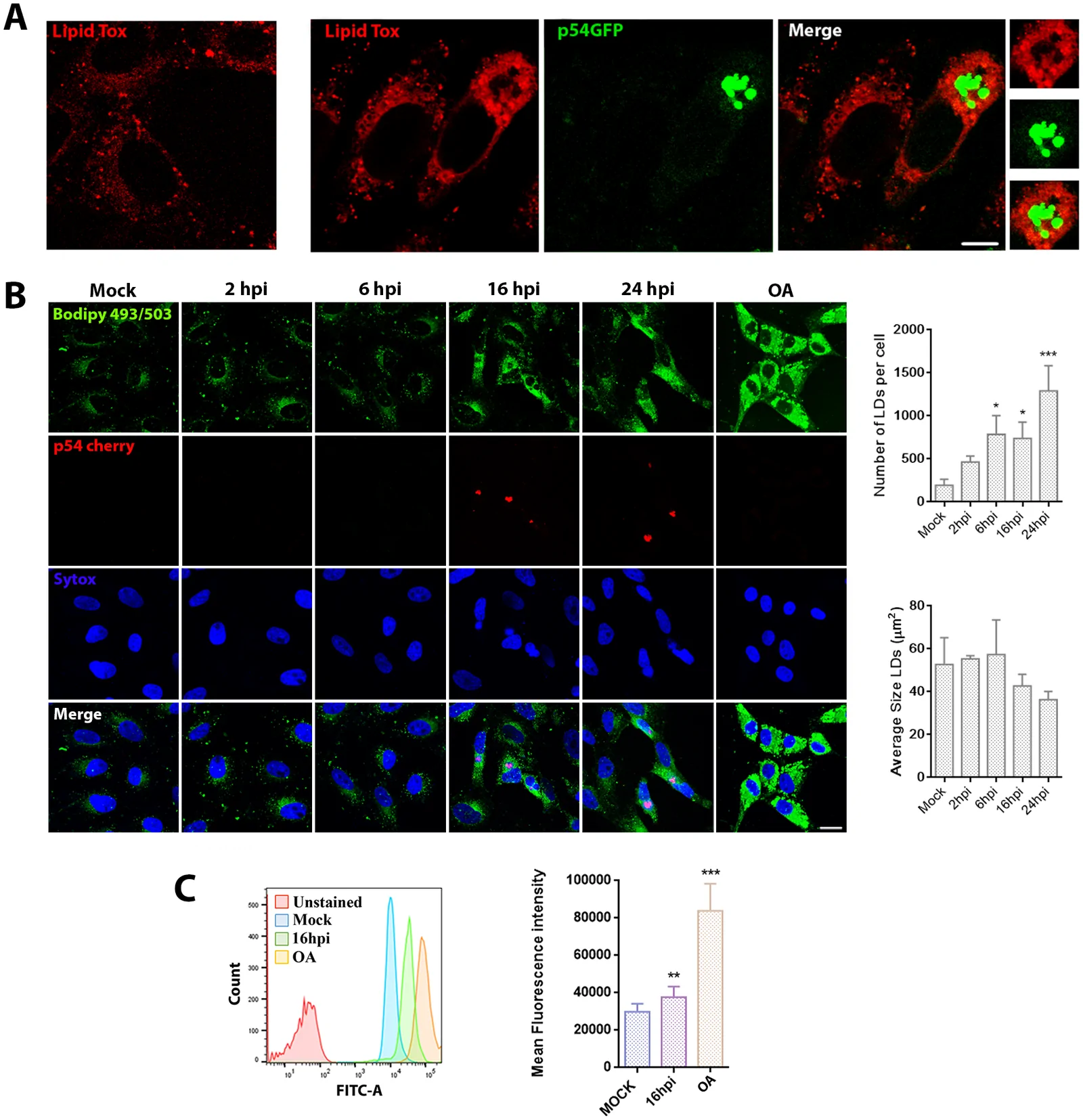
**(A)** Redistribution of LDs in ASFV infected cells. Mock and ASFV infected Vero cells were stained for LDs using LipidTOX dye. Cells treated with OA were included as a positive control. Viral factories were detected using a recombinant ASFV expressing the p54 viral protein fused to GFP. Scale bar: 20 µm. **(B)** LD staining using BODIPY dye at different time points during ASFV infection. Nuclei are labeled with SYTOX. Cells treated with OA were included as a control. Viral factories were detected using a recombinant ASFV expressing the p54 viral protein fused to mCherry. The scale bar (20 µm) shown in the representative panel applies to all images. The number of LDs per cell and the mean LD area were determined following threshold-based segmentation of BODIPY-positive structures. **(C)** Representative overlay histograms of BODIPY fluorescence measured by flow cytometry are shown. Flow cytometric analysis enabled a statistical comparison of LD abundance between infected and uninfected cells. The bar graph represents the quantification of the histogram data. Statistically significant differences are indicated by asterisks (*p < 0.05; **p < 0.01; ***p < 0.001).

LipidTOX was used to visualize LDs in virus-infected cells at 16hpi by confocal microscopy. While LDs appeared as small, scattered dots in uninfected cells, after infection the fluorescent signal reorganized into ring-shaped structures surrounding sites of viral assembly, which were identified as viral factories by staining for the viral protein p54 (**Figure 3A**). Confocal fluorescence imaging and flow cytometry were used to detect the presence of LDs using BODIPY 493/503, another dye commonly used to stain cellular LDs. Cells treated with oleic acid (OA) acted as positive controls due to its robust induction of LD formation. A substantial increase in LD abundance was observed as viral infection progressed (**Figure 3B**). Flow cytometry results further confirmed the higher LD signal in infected cells compared to the uninfected cells (**Figure 3C**).

### 3.5 Inhibition of lipid droplet biosynthesis prevents ASFV replication

**Figure 4:**
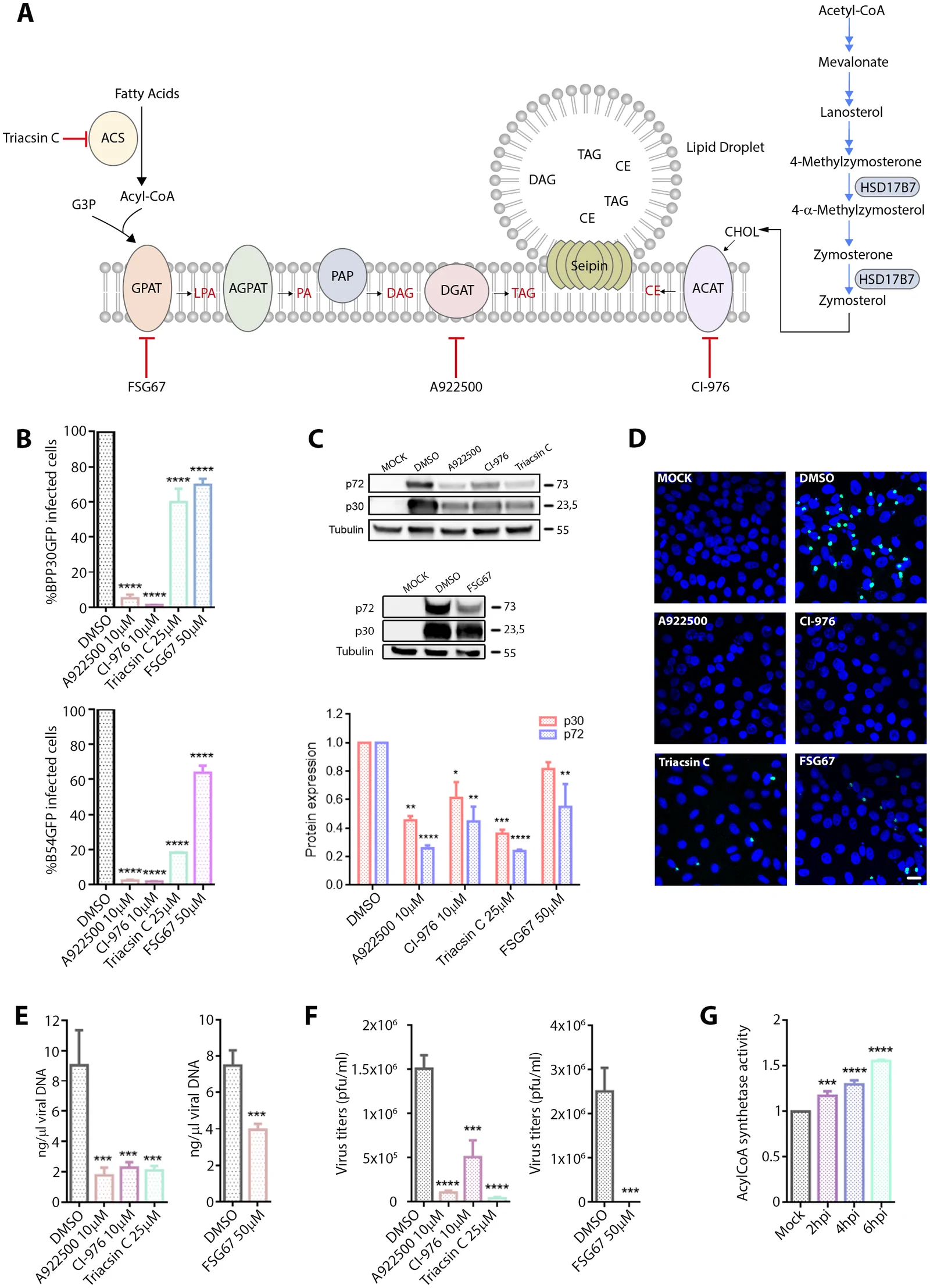
**(A)** Schematic representation of LD biosynthesis. ACS, acyl-coenzyme A synthetase; GPAT, glycerol-3-phosphate acyltransferase; AGPAT, 1-acylglycerol-3-phosphate O-acyltransferase; PAP, phosphatidate phosphatase; DGAT, diacylglycerol acyltransferase; ACAT, acyl-CoA:cholesterol acyltransferase; HSD17B7, 3-keto-steroid reductase/17β-hydroxysteroid dehydrogenase 7; G3P, glycerol-3-phosphate; LPA, lysophosphatidic acid; PA, phosphatidic acid; DAG, diacylglycerol; TAG, triacylglycerol; CHOL, cholesterol; CE, cholesteryl ester. Inhibitors used in this study are indicated in red. **(B)** Infection percentages of ASFV BPP30GFP and B54GFP expressing viruses at 16 hpi were determined by flow cytometry in DMSO treated control cells or in Vero cells treated with the indicated inhibitors. **(C)** Immunoblot analysis of viral proteins expression at 16 hpi in mock and ASFV infected treated cells with the indicated inhibitors. Tubulin was used as a loading control. Densitometric quantification of protein levels is shown below. **(D)** Representative confocal images of Vero cells treated with the indicated inhibitors and infected with B54GFP for 16 hpi. Nuclei are stained in blue, and viral factories are identified by GFP signal. The scale bar (20 µm) shown in the representative panel applies to all images. **(E)** ASFV genome copy number quantified by real-time qPCR. **(F)** Production of infectious virus determined by plaque assay. Percentages were normalized to values obtained from DMSO treated cells. **(G)** Modulation of acyl CoA synthetase activity in ASFV-infected cells. Data were normalized to the enzymatic activity measured in lysates from mock-infected cells. Asterisks indicate statistically significant differences (*p < 0.05; **p < 0.01 ***p < 0.001; ****p < 0.0001).

LD biogenesis occurs in the endoplasmic reticulum (ER) and is facilitated by a series of sequential reactions catalyzed by specific enzymes and metabolic intermediates (**Figure 4A**). To validate these factors as potential antiviral targets, we examined their functional relevance during ASFV infection using specific inhibitors of LD biosynthesis. A922500 is a DGAT-1 inhibitor (Dias et al., 2020), whereas CI-976 is a potent and specific inhibitor of ACAT (Krause et al., 1993). Cells were pretreated with the indicated compounds 1 h before infection, and treatments were maintained throughout the infection cycle using previously established non-toxic concentrations (**Supplementary Figure 2**). Subsequently cells were infected with ASFV BPP30GFP or B54GFP and infected cells were quantified by flow cytometry. Both inhibitors significantly reduced ASFV infectivity by 95–99%, with IC50 values of 1.42 µM (A922500) and 7.13 µM (CI-976), respectively.

Triacsin C, a potent inhibitor of long-chain fatty acyl-CoA synthetase (ACSL1) (Tomoda et al., 1991; Matsuda et al., 2008), also exhibited antiviral activity consistent with preliminary findings from our laboratory (Garcia-Dorival et al., 2023). Treatment with Triacsin C reduced ASFV infectivity by approximately 40% compared with DMSO-treated controls (IC50 ≈ 25 µM; **Figure 4B**). FSG67, an inhibitor of GPAT (Clemens et al., 2019), significantly reduced BPP30GFP infectivity to 20–30%. Infectivity of ASFV B54GFP was further assessed in the presence of the inhibitors, yielding similar results, except ca. 80% reduction in cells treated with Triacsin C.

The reduction in infectivity caused by fatty acid biosynthesis inhibitors correlated with decreased expression levels of the early viral protein p30 and the late protein p72, as determined by western blotting in BA71V-infected cells under the same experimental conditions as described above (**Figure 4C**). Furthermore, a marked reduction in the number of viral factories was observed in B54GFP-infected cells in the presence of A922500, CI-976, or Triacsin C after 16 h, whereas only a slight decrease was detected in FSG67-treated cells, as assessed by confocal microscopy. (**Figure 4D**).

Finally, the presence of these inhibitors during infection also reduced viral DNA synthesis at 16 hpi, as quantified by RT-qPCR (**Figure 4E**), and significantly decreased viral production at 24 hpi (**Figure 4F**). Collectively, these results strongly indicate that LDs constitute critical organelles for efficient ASFV replication.

### 3.6 Acyl-CoA synthetase activity in infected cells is stimulated early at infection

Next, we measured ACS activity in lysates from Vero infected cells at several times post infection. Infected cells exhibited an increase in ACS activity as early as 2 hpi, and this activity continued to rise steadily during the initial phases of infection (**Figure 4G**). These findings indicate that ACS activity was stimulated from very early stages post-infection.

### 3.7 Viral proteins and viral DNA are associated with LDs

**Figure 5:**
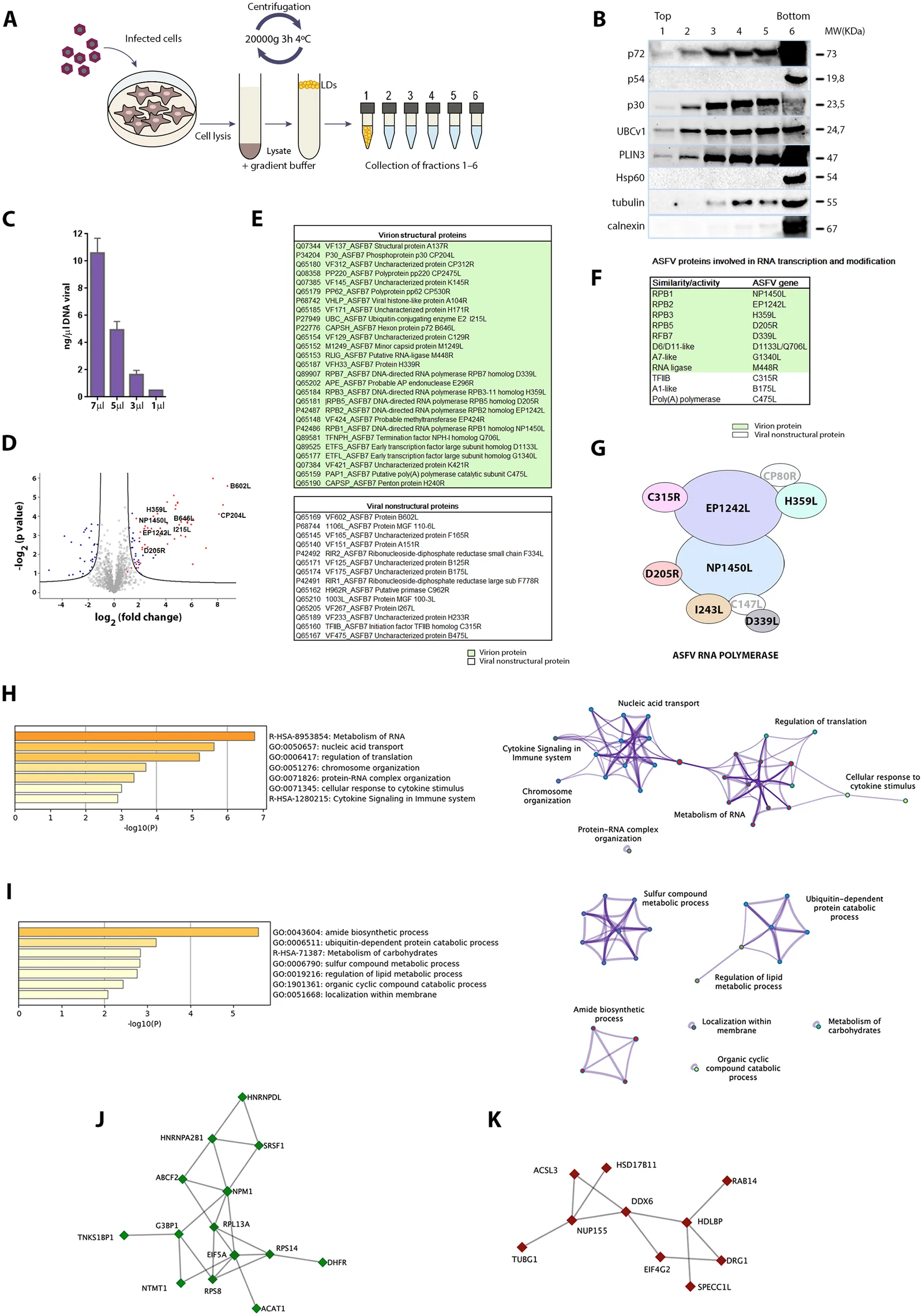
**(A)** Schematic representation of the LD purification procedure. **(B)** Immunoblot analysis of ASFV proteins (p72, p54, p30 and UBCv1) and cellular proteins in LD-enriches fraction (1) and non-floated fractions (2-6) after purification of LDs from infected cells. Calnexin, tubulin and Hsp60 were included as purity controls for the endoplasmic reticulum, cytoplasm and mitochondria, respectively. **(C)** Detection of viral DNA by quantitative real-time PCR using increasing volumes (1, 3, 5, and 7 µl) of fraction 1 (LD-enriched fraction). The X-axis indicates the volume of fraction analyzed. **(D)** Volcano plot showing proteins identified in the LD proteome of non-infected versus ASFV-infected cells. Differentially enriched viral proteins are highlighted in red and some of them labeled; significantly regulated host proteins are shown in blue, whereas gray points represent proteins without significant changes. **(E)** Table listing viral proteins detected in the LD-associated proteome. Virion structural proteins are highlighted in green and viral non-structural proteins in white. **(F)** Viral proteins involved in RNA transcription and modification identified within the LD proteome. **(G)** Schematic representation of ASFV RNA polymerase (adapted from Rodríguez J.M. et al. 2013). **(H, I)** Bioinformatic functional enrichment analysis of proteins enriched **(H)** and depleted **(I)** in the LD proteome. Bar graph represents the top seven enriched terms (such as GO terms and metabolic pathways) colored according to their -log10 (*p*-value) **(J, K)** Protein-protein interaction networks of host proteins enriched **(J)** and depleted **(K)** in LD fractions from ASFV-infected cells.

We purified LDs from ASFV-infected Vero cells using a flotation protocol (**Figure 5A**) and examined the presence of viral proteins associated with these organelles by immunoblotting. In the top LD enriched fraction, we detected the viral proteins p30, p72, and UBCv1, indicating that these viral components may associate with LDs. In contrast, the viral protein p54 was absent from the LD enriched fraction but was readily detected in the non-floated fractions, which also contained tubulin, calnexin, and Hsp60-markers of the cytoplasm, ER membrane, and mitochondria, respectively (**Figure 5B**).

We further investigated whether viral DNA was present in the LD-enriched fraction. Serial dilutions of the purified LD fraction were analyzed by quantitative PCR. The results suggested a potential association between viral DNA and the LD enriched fraction (**Figure 5C**).

### 3.8 ASFV infection induces changes in the expression of lipid droplet-associated proteins

We performed a proteomic analysis in the purified LDs. To visualize the global distribution of proteins enriched in the LD fraction, a volcano plot was generated, displaying differentially expressed proteins. In this plot, red dots represent significantly enriched viral proteins, blue dots denote significantly enriched cellular proteins, and gray dots correspond to proteins without significant changes (**Figure 5D**).

Of the ASFV encoded 151 proteins, 41 were identified in association with LDs, including viral proteins UBCv1, p72, and p30, previously detected in this fraction by immunoblotting. Notably, 27 of the 41 LD-associated viral proteins were structural components of the ASF viral particle, while the remaining 14 were non-structural proteins (**Figure 5E**). Importantly, 12 of the 22 ASFV proteins known to participate in RNA transcription and modification, including multiple subunits of the viral RNA polymerase (**Figure 5G**) and specific transcription factors (**Figure 5F**), were enriched in the LD proteome. In contrast, the remaining 10 ASFV proteins involved in RNA processing and polyadenylation were absent from the LD fraction.

ASFV infection also altered the profile of cellular proteins associated with LDs. We identified 20 proteins present in LDs from infected cells that were not detected in those of non-infected cells. Functional enrichment analysis performed with Metascape revealed significant enrichment in terms related to RNA metabolism and nucleic acid transport. In addition, pathways related to cytokine signaling and cellular responses to external stimuli were also enriched, suggesting that ASFV infection impacts both gene expression regulation and host immune related processes (**Figure 5H**). Metascape analysis further identified pathway associations, predicted protein-protein interaction networks, and grouped significant proteins into functional clusters, including those involved in the regulation of lipid metabolic processes.

Conversely, 23 proteins were detected in LDs from non-infected cells but not in LDs from infected cells. Functional enrichment analysis of this group of proteins indicated associations with metabolic and catabolic pathways, including amide biosynthetic process, ubiquitin-dependent protein catabolism, sulfur compound metabolism, and lipid metabolic regulation (**Figure 5I**), indicating a potential reduction in host metabolic and protein turnover processes within the LD fraction upon infection. Notably, this group included several ribonucleoproteins, ribosomal proteins, ACAT1, and additional enzymes involved in lipid metabolism. Protein-protein interaction network analysis further revealed distinct functional clusters among proteins both enriched and depleted in the LD fraction upon ASFV infection. Proteins enriched in infected samples formed a highly interconnected network centered on ribosomal proteins and RNA binding factors, including RPS14, RPS8, EIF5A and members of the HNRNP family, suggesting an increased association of translation and RNA processing related components with LDs in infected samples (**Figure 5J**). In contrast, proteins depleted from the LD fraction displayed a less interconnected network, comprising factors involved in vesicular trafficking and lipid metabolism, such as RAB14, ACSL3, and HSD17B11 (**Figure 5K**), consistent with a reduced association of lipid metabolic and intracellular transport functions.

## 4 Discussion

Transcriptomic and proteomic approaches have accelerated the exploration of complex host-pathogen interactions. Advances in next-generation sequencing enabled us to perform a transcriptomic analysis of porcine macrophages. This study focused on comparing transcript profiles between ASFV-infected and uninfected macrophages at 6 and 16 hpi. At 6 hpi, the incoming virus has completed cell entry and early viral protein expression; however, viral genome replication and late protein expression have not yet occurred. In contrast, by 16 hpi, viral genome replication and late protein expression have been completed, corresponding to the first round of viral replication. Transcriptomic analysis revealed that ASFV modulated multiple host metabolic pathways while suppressing components of the host immune response. This modulation was associated with activation of antiviral signaling pathways, including the induction of interferon stimulated genes, as well as genes involved in inflammatory and cytokine responses, such as TNF and CCL4. Furthermore, transcriptomic analyses uncovered significant regulation of genes associated with apoptosis and autophagy following ASFV infection. These findings are consistent with previous studies describing the complex molecular mechanisms underlying host apoptotic responses to ASFV infection (Wu et al., 2021), further supporting and strengthening our findings.

Remarkably, we observed an upregulation of genes involved in Lipid Droplets (LDs) biosynthesis, a central pathway of lipid metabolism and multiple cellular processes, further validated by RT-PCR analysis. LDs are dynamic cellular organelles involved in the storage and mobilization of triacylglycerols and cholesteryl esters. These organelles are essential for lipid availability and membrane biosynthesis, yet their functional importance has been largely underappreciated (Ricciardi et al., 2022; Viktorova et al., 2018). This finding was particularly relevant given that lipids are essential for ASFV infection and replication, and recent evidence indicates that ASFV actively exploits LD metabolism by inducing LD accumulation, altering LD lipid composition, and promoting replication through lipolysis-dependent lipid mobilization, moreover, oleic acid-induced LD biogenesis has been shown to enhance ASFV replication in a dose-dependent manner (Yang et al., 2026). This virus induces intracellular accumulation of total cholesterol (CHOL) and triglycerides (Chen et al., 2023; Cuesta-Geijo et al., 2015). CHOL flux plays a critical role at early infection in the establishment of a productive infection, as ASFV actively remodels intracellular CHOL trafficking by enhancing cellular uptake and redistributing free CHOL to viral replication sites (Cuesta-Geijo et al., 2015; Galindo et al., 2019). In addition, ASFV infection disrupts lipid and glucose metabolism and promotes the upregulation of host genes associated with lipid biosynthesis (Chu et al., 2024).

Enzymes involved in LD biosynthesis were significantly upregulated in infected cells included ACAT1, DGAT1, ACSM5, and seipin. The early induction of ACSM5 and DGAT1 indicated that ASFV rapidly enhanced fatty acid activation and triacylglycerol synthesis shortly after infection, possibly to ensure LD availability; in contrast, ACAT1 and BSCL2 were induced at later stages, consistent with the role of these enzymes in CHOL esterification and LD growth and maturation. The upregulation of HSD17B7 further supported enhanced CHOL biosynthesis during infection. In addition to HSD17B7, our RNA-seq data showed differential regulation of additional mevalonate/cholesterol pathway genes, including upregulation of HMGCR (3-hydroxy-3-methylglutaryl-CoA reductase) and downregulation of HMGCS1 (3-hydroxy-3-methylglutaryl-CoA synthase 1). These results support the view that ASFV modulates host cholesterol. In line with recent observations, sterol metabolism tracks with ASFV growth and constrains replication when inhibited (Chu et al., 2024). In fact, the inhibition of DGAT1 (A922500) and ACAT (CI-976) produced strong antiviral effects (>95% reduction in infectivity), consistent with their central roles in neutral lipid synthesis; inhibition of ACSL1 (Triacsin C) or GPAT (FSG67) also reduced replication, in keeping with the idea that fatty acid activation and glycerolipid initiation could feed essential lipid pools during infection. Interestingly, FSG67 almost completely abolished infectious viral production despite only partially reducing viral DNA levels. This suggests that FASN inhibition may affect not only viral replication but also lipid-dependent steps involved in virion assembly, maturation, or infectivity, resulting in a stronger impact on infectious virus yield.

Previously, we had identified Triacsin C as a promising inhibitor of ASFV infection (Garcia-Dorival et al., 2023). Moreover, this compound demonstrated antiviral activity against several other viruses, including human cytomegalovirus (HCMV), rotavirus, hepatitis viruses and SARS-CoV-2, highlighting the importance of this branch of lipid metabolism for diverse viral pathogens (Koyuncu et al., 2013; Kim et al., 2012; Liefhebber et al., 2014; Santos-Beneit et al., 2021; Yang et al., 2026). In addition, inhibitors targeting enzymes involved in LD biogenesis have previously been shown to impair the replication of norovirus (Chang, 2009), rotavirus (Crawford and Desselberger, 2016), hepatitis C virus (HCV) (Hu et al., 2017) Herpes simplex virus-1, influenza A, dengue virus and zika virus (Monson et al., 2021), further supporting the relevance of neutral lipid synthesis in viral infection cycles.

An increase in LD abundance upon infection was demonstrated by both confocal microscopy and flow cytometry. Morphometric analysis revealed an increase in LD number during infection, whereas mean LD area remained largely unchanged, indicating that the increased BODIPY fluorescence primarily reflects greater LD abundance rather than larger droplets. Confocal microscopy additionally revealed the redistribution of LDs toward viral assembly sites. This spatial redistribution suggests that LDs are not merely passively accumulating during infection, but may be actively recruited to support viral replication and morphogenesis. LD recruitment to assembly organelles has been described for several other viruses and is thought to facilitate membrane supply, provide a local energy source, and act as a macromolecular scaffold that supports the organization of viral processes (Herker, 2024). Beyond their established role in lipid storage and metabolism, LDs are increasingly recognized as dynamic hubs that integrate metabolic and innate immune responses during viral infection. In our study, the observed increase in LD abundance upon infection, together with the upregulation of the interferon-stimulated gene viperin in the transcriptomic analysis, supports the notion that LDs may participate in the antiviral response. LDs have been described as sites for viperin localization, and this association is important for optimal antiviral activity (Hinson and Cresswell, 2009). Moreover, viperin exerts broad-spectrum antiviral activity against diverse viruses, including flaviviruses, hepatitis C virus, cytomegalovirus, and influenza virus, through mechanisms involving disruption of lipid metabolism and inhibition of viral replication complexes (Helbig and Beard, 2014). In these viral infections, remodeling of LDs may contribute not only to alter cellular lipid homeostasis but also to the sabotage of antiviral defenses.

Furthermore, the rapid activation of acyl CoA synthetase activity within the early times of infection reinforces the idea that ASFV initiates an early metabolic shift to sustain lipid flux and possibly accommodate the demands of viral factory assembly. This is consistent with previous studies showing that infection by other viruses also induces the upregulation of long-chain acyl-CoA synthetase activity to support membrane biogenesis and replication organelle formation (Nchoutmboube et al., 2013).

Proteomic characterization of purified LDs provided evidence that these organelles serve as platforms for viral component recruitment. We identified 41 ASFV proteins associated with LD fractions, including structural proteins and nonstructural proteins. Notably, we detected numerous viral factors involved in RNA transcription and modification, including putative subunits of the viral RNA polymerase and transcription factors (Rodriguez and Salas, 2013), similar to other viruses where LDs themselves scaffold replication/assembly intermediates (Qin et al., 2022). Together with the detection of viral DNA in purified LD fractions, these results support the hypothesis that LDs may contribute to processes related to viral ASFV RNA transcription. However, no infectious virions were detected in the purified LD preparations suggesting that late expression genes transcription and new progeny assembly occurs in different areas of viral factories. Accordingly, ASFV infection also reshaped the host LD proteome, enriching cellular proteins involved in RNA metabolism, nucleic acid transport, and lipid regulation, while LDs from uninfected cells were relatively enriched for amide biosynthesis, ribonucleoproteins, and ribosomal proteins.

In conclusion, ASFV infection induces significant alterations in host gene expression, including genes involved in LD biosynthesis. Pharmacological inhibition of LD formation results in a marked impairment of ASFV replication. Notably, both structural and non-structural viral proteins were identified within the LD associated proteome. Collectively, these findings underscore the substantial impact of ASFV on host lipid metabolism, provide a framework for further investigation into virus–host interactions mediated by cellular lipid pathways, and point to potential targets for antiviral intervention.

## Data Availability

The datasets presented in this study can be found in online repositories. The names of the repository/repositories and accession number(s) can be found below: https://doi.org/10.5281/zenodo.19567515, https://doi.org/10.5281/zenodo.19565956.

## Supplementary material

Supplementary Figure 1 Primer sequences used for qPCR in Vero cells and porcine alveolar macrophages.

Supplementary Figure 2 Cell viability of the reagents used in this study. Based on these results, the CC50 values for each compound were calculated, and optimal non-toxic working concentrations were selected for subsequent infection assays.
